# Evaluation and guide for embedding opioid use disorder education in health professions’ curricula

**DOI:** 10.1186/s12909-023-04088-5

**Published:** 2023-03-01

**Authors:** Julie G. Salvador, Sindy L. Bolaños-Sacoman, Joanna G. Katzman, Ann E. Morrison, Lindsay E. Fox, Jennifer S. Schneider, Snehal R. Bhatt, Tyler W. Kincaid, V. Ann Waldorf

**Affiliations:** 1 Health Sciences Center, University of New Mexico, 1 UNM, Albuquerque, NM MSC09 5030 USA; 2 SBS Evaluation & Program Development Specialists, LLC, Albuquerque, NM USA

**Keywords:** Buprenorphine training, Opioid treatment, Health professional training

## Abstract

**Background:**

Morbidity and mortality from Opioid Use Disorder is a health crisis in the United States. During the COVID-19 pandemic, there was a devastating increase of 38.4% in overdose deaths from the 12-month period leading up to June 2019 compared with the 12-month period leading up to May 2020, primarily driven by synthetic opioids. Buprenorphine is an effective medication for opioid use disorder but uptake is slow due in part to lack of provider knowledge, confidence, and negative attitudes/stigma toward patients with OUD. Addressing these barriers in academic training is a promising approach to building workforce able to effectively treat opioid use disorder.

**Methods:**

Our university developed a training for pre-licensure physicians, physician assistants and psychiatric nurse practitioners that included the DATA Waiver training and a shadowing experience. Expected outcomes included improved knowledge, skills and attitudes about persons with OUD and buprenorphine treatment, plans to provide this treatment post-graduation, for pre-licensure learners to have completed all requirements to prescribe buprenorphine post-graduation, and for the training to be embedded into school’s curricula.

**Results:**

Results were positive overall including improved knowledge and attitudes toward persons with OUD, better understanding of the benefits of this treatment for patients, increased confidence and motivation to provide this treatment post-graduation. The training is now embedded in each program’s graduation requirements.

**Conclusion:**

Developing a didactic and experiential training on buprenorphine treatment for opioid use disorder and embedding it into medical, physician assistant, and psychiatric nurse practitioner licensure programs can help prepare future providers to treat opioid use disorder in a range of settings. Key to replicating this program in other university settings is to engage faculty members who actively provide treatment to persons with OUD to ensure shadowing opportunities and serve as role models for learners.

**Supplementary Information:**

The online version contains supplementary material available at 10.1186/s12909-023-04088-5.

## Background

Opioid use disorder (OUD) remains a public health crisis in the United States (U.S.). In 2020, nearly 92,000 people in the U.S. died from a drug overdose and 75% of these (68,630 deaths) involved an opioid [1]. During the COVID-19 epidemic, overdoses surged in the U.S. Compared to the previous year, overall drug overdose deaths increased almost 30% in 2020, exceeding 90,000. Alarmingly, recent provisional data from the Centers for Disease Control and Prevention show over 100,000 deaths in the U.S. between April 2020 and April 2021 an increase of nearly 30% from the prior 12 month period [2]. Treatment for opioid use disorder (OUD) using FDA approved medications including methadone, buprenorphine or naltrexone, referred to as Medications for Opioid Use Disorder (MOUD), is the standard of care [3, 4]. Methadone is highly regulated and can only be prescribed from Opioid Treatment Programs (OTPs), limiting access to this medication, particularly in rural and underserved areas. Buprenorphine is a particularly effective medication that can be easily provided to patients by physicians (Medical Doctors (MD) and Doctors of Osteopathic Medicine (DO)), nurse practitioners (NPs), physician assistants (PAs) and other eligible advanced nursing specialist providers in primary care settings, helping to expand access. However, despite its clear benefit, uptake of this MOUD has been slow, and treatment need still outweighs access [5]. Barriers to the adoption of MOUD include factors such as lack of provider knowledge, negative attitudes/stigma toward patients with OUD, policy and regulatory constraints and concerns with financial reimbursement to support start up and sustainability of buprenorphine treatment [6, 7].

Historically, physician training has not focused on preparing future providers to treat opioid use disorder. Therefore, to address the opioid crisis in the U.S., already practicing physicians need to be identified and encouraged to participate in buprenorphine training and to begin this treatment with patients with OUD. While this approach has helped to increase availability of treatment nationally, there are still many providers who decide not to pursue the training and start this treatment. In fact, there remains a shortage of primary care providers waivered to prescribe buprenorphine for OUD in the U.S. [8] and most rural counties do not have any waivered provider [9]. Practicing providers who did not learn this in school and were not exposed to working with persons with OUD, may often feel too unprepared and uncomfortable starting this treatment. Therefore, initiatives that start this education as a part of the standard curriculum required for medical school graduation can go a long way toward increasing access [10].

To address barriers to starting buprenorphine treatment and help prepare future providers to treat OUD, a buprenorphine training initiative for interprofessional pre-licensure physicians, physician assistants and psychiatric nurse practitioner students was developed and implemented at the University of New Mexico Health Sciences Center, focused on medical, physician assistant students, and psychiatric nurse practitioner students. This training, called the *University Providers Clinical Support System Training Initiative (U PCSS-TI)* was embedded into each program’s standard curricula required for graduation. Core program components of the intervention included the required DATA Waiver training and an in-person shadowing experience. The DATA waiver component is the didactic portion that includes an overview of OUD and how to initiate treatment, including details of prescribing, dosing and monitoring. The shadowing component provides first-hand exposure to this treatment in a clinical setting, alongside an expert buprenorphine practitioner. Post-graduation, students are encouraged to join an online ECHO telementoring program that provides ongoing education and support for starting buprenorphine treatment in practice. An evaluation of the intervention’s impact on learners’ attitudes and perceived knowledge and skills was conducted. The purpose of this manuscript is to describe our initiative and evaluation in order to help other institutes of higher education to develop similar interventions that can be successfully embed into their standard training curricula.

## Methods

The aim of this evaluation is to determine the impact of the U PCSS-TI on learner’s perceptions about and knowledge of OUD and its treatment using FDA approved medications with focus on buprenorphine, confidence in their ability to provide this treatment, and interest and motivation to implement this treatment post-graduation. A federally required survey of satisfaction with the training was also collected and reported herein. The evaluation uses a single group, post-test design. The intervention and study was conducted at the University of New Mexico Health Sciences Center. The target population for the intervention and study were the current cohort of pre-licensure students in the UNM Medical School, Physician Assistant program, and Psychiatric Nurse Practitioner program. All participants in this intervention were pre-licensure, referred throughout this manuscript as ‘students’ or ‘learners.’

### Step 1: Selecting and collaborating with intervention Leadership and Faculty Champions

In the present study and for other higher education settings to consider, the choice of key Faculty members and Administrators is the first step in developing the intervention, implementation, and evaluation. These leaders play vital roles in determining how to modify existing curriculum to include main intervention components (DATA waiver training and shadowing), ensuring learners participate in the training and shadowing experiences (scheduling), ensuring the intervention is embedded permanently into program curricula (sustainability), and ensuring learner’s complete evaluation surveys. Faculty member champions selected had expertise in OUD treatment, MOUDs, and held positions of influence in the pre-licensure programs (the School of Medicine, which houses the Physician Assistant program and the College of Nursing which houses the Psychiatric Mental Health Nurse Practitioner program). Faculty member champions and other members of the study team (lead evaluator, consultant, administrative support, and study Principal Investigator) agreed to monthly meetings during the entire implementation and study period to ensure smooth intervention development, implementation, data collection and to address any barriers. Table 1 provides details on these faculty members’ roles within the university that allowed for the successful development, rollout and sustainability of this training program. Additionally, the study coordinator and research team (PI, lead evaluator, study consultant) met weekly to develop training schedules, coordinate shadowing opportunities, and review data collected.

**Table 1:** UNM PCSS-TI Leadership and Roles

POSITION	ROLE
*Vice Chair of Behavioral Sciences, DPBS*. Associate Professor and Vice Chair for the Department of Psychiatry and Behavioral Sciences (DPBS) and Associate Professor, Department of Family and Community Medicine	Principal Investigator/Project Director. Ensured strong collaborations with educational faculty members across the School of Medicine (SOM). Clinical expertise working with persons with substance use disorders ensures first-hand knowledge of Opioid Use Disorder (OUD) and Medications for Opioid Use Disorder (MOUD).
*Chief of Addictions, DPBS*. This faculty member is board-certified in addictions and psychiatry and is acting Chief of Addictions at the DPBS. The Clinical Trainer for the University PCSS project is the Director at UNM Addiction and Substance Abuse Program (ASAP). ASAP is a substance use treatment clinic through University of New Mexico Hospital and is staffed by faculty members from the Department of Psychiatry and Behavioral Sciences.	Provided MOUD treatment for persons with substance use disorders at the University of New Mexico (UNM) ASAP. He is a certified Drug Addiction Treatment Act (DATA) Waiver trainer with years of training experience. This position ensured the full DATA waiver training was provided at flexible times, helping ensure it could be easily embedded into the existing curriculum structure. This faculty member also was able to provide the shadowing component through his work at the ASAP clinic, one of Albuquerque’s largest Opioid Treatment Programs.
*Director, UNM Pain Center, UNM Health Sciences Center (HSC).* This faculty member is a neurologist and specializes in treatment of chronic pain. She has a long history of working with persons with OUD and expanding access to naloxone statewide.	Given the position of this faculty member at the Pain Center ensured that students had opportunities to shadow clinicians providing OUD treatment in the context of chronic pain treatment.
*Inter-Professional Education Coordinator, UNM HSC.* This faculty member is a board-certified internal medicine specialist responsible for a major component of the medical school curricula and for promoting collaboration between and within different health professions.	This faculty member’s position in the medical school ensured the training initiative was embedded into the pre-clinical medical school curriculum. Additionally, she provided the opportunity to shadow MOUD treatment in her office-based medical practice.
*Director, Medical Student Education, HSC*. This faculty member is an Associate Professor in the Department of Psychiatry and Behavioral Sciences in the School of Medicine and a board-certified Psychiatrist and the Psychiatric Clerkship Director.	This position played an integral role in ensuring the DATA Waiver training was embedded and sustained in the school of medicine. Furthermore, she ensured that the clinical experience was embedded in the Psychiatry clerkship, thereby allowing medical students to participate in the shadowing experience.
*Clinical and Outreach Coordinator with the UNM Physician Assistant program*. This faculty member is the lead for the PA Program at the HSC and provides direct OUD treatment at the HSC Family and Community Medicine Clinic.	This position ensured that all PA learners received the DATA waiver training and were placed in a shadowing experience alongside faculty members in their same academic discipline during clinical rotations. Furthermore, this faculty member obtained the DATA waiver training certification to provide students with trainers from both MD and PA backgrounds.
*Assistant Professor and Concentration Coordinator for the Psychiatric Nurse Practitioner program, HSC, College of Nursing.* This faculty member is the Coordinator of the Psychiatric Mental Health Nurse Practitioner (PMHNP) Program at the College of Nursing. She oversees student clinical placements.	Given this faculty member’s position as faculty, she acted as lead for coordinating the DATA waiver training and embedding the PCSS program into the curriculum for psychiatric nurse practitioners.

Acronyms: Department of Psychiatry and Behavioral Sciences (DPBS); University of New Mexico (UNM); School of Medicine (SOM); Health Sciences Center (HSC); Drug Addiction Treatment Act (DATA); Physician Assistant (PA), Psychiatric Mental Health Nurse Practitioner (PMHNP), Medical Doctor (MD); Opioid Use Disorder (OUD); Medications of Opioid Use Disorder (MOUD); Addiction and Substance Abuse Program (ASAP).

### Step 2: Determining and implementing intervention components

The main components of the **U PCSS-TI** training program were (1) the federally required and approved 8-hour DATA waiver training and (2) an experience shadowing a trained OUD treatment provider in practice. Having the intervention contain both the DATA waiver training and shadowing was designed to focus on major categories in Blooms taxonomy of learning [11]. The DATA waiver training reflects the first level of knowledge and the shadowing reflects the later categories that focus on skills, abilities, comprehension and application. Participation in the ECHO to help clinicians start this treatment once they are in practice was encouraged but not a required program component.

*DATA waiver training*. Completion of the DATA waiver training was the first phase of the University PCSS intervention. This training component is 8 h for MDs and 24 h for NPs and PAs. The DATA waiver training includes a required ‘live’ component that can be either 4 or 8 h, and can be completed in person or via zoom. In our intervention, we offered both the 4 and 8 h live training options and the faculty member champions decided which length best fit into their curriculum. Any additional DATA waiver educational modules needed to meet the full 8 h for MDs or 24 h for NPs/PAs were required to be completed by learners as a graduation requirement (at some point prior to graduation). NPs and PAs are required to complete additional online modules due to their different training and licensure backgrounds compared to MDs. The ‘live’ portion (4 or 8 h) is identical for all prescribers (MDs, NPs and PAs).

*Shadowing*. A four-hour clinical shadowing experience was provided at four sites: the University Pain Center, the University Hospitals’ Addiction and Substance Abuse Programs, a local Opioid Treatment Program, and a local Family Community Medicine primary care clinic. All sites had providers with expertise and willingness to allow the clinical observation of treatment intake, screening, counseling, and buprenorphine inductions, at a minimum. Several sites also allowed students to observe the prescribing of additional medications for co-occurring disorders, addressing needs of special populations, two of which were youth and pregnant/postpartum women. Students also witnessed the utilization of the prescription monitoring program to understand how to address the potential for aberrant behaviors such as misuse, overuse or diversion. Finally, students were trained in the importance of co-prescribing of naloxone rescue kits and medication management. Table 2 shows the sites selected to provide students a variety of shadowing opportunities in which to participate.

*Post training and shadowing support*. Post shadowing, students were and continue to be encouraged to participate in an ECHO program focused on medications for opioid use disorder, focused on buprenorphine. Using the Extensions for Community Healthcare Outcomes model [12] this ECHO provides ongoing education regarding buprenorphine-based OUD treatment, an opportunity to learn from providers practicing this treatment, and a venue for graduates to bring de-identified patient cases for presentation to support their implementation in professional clinic settings. Details of this MOUD ECHO have been published [13].

**Table 2:** Shadowing locations

Shadowing Site	Patient population/setting
UNM Pain Center	Chronic pain; academic setting
UNM Hospitals’ Addiction and Substance Abuse Programs (ASAP)	Comprehensive substance use treatment clinic with an Opioid Treatment Program; academic setting
Recovery Services of New Mexico	Opioid Treatment Program; community-based setting
Southwest Mesa Clinic	Primary care; academic-affiliated community setting

### Data collection and analysis

Three survey instruments were used to collect data from learners regarding the impact of the intervention focused on changes in attitudes, confidence and perceived knowledge in providing this treatment and plans to provide it in practice after graduation. These are described below. All data was collected by the study evaluator between April 1, 2018 through September 29, 2021 using Survey Monkey, an online data collection platform. The first two surveys were collected immediately following the DATA waiver training. All learners including MD, NPs and PAs had the same ‘live’ DATA waiver training. The training satisfaction and personal impact surveys were all completed post DATA waiver ‘live’ component (the zoom-based or in-person part of the DATA waiver training and prior to any shadowing exposure. The third survey was completed within thirty days following the learner’s shadowing experience. Data from the three surveys were analyzed using descriptive statistics. All surveys were anonymous. Informed consent was obtained from all students via an online consent form prior to completing data collection. Internal reliability using Cronbach’s Alpha was assessed for all three surveys and is included in the results.

- SAMHSA’s Government Performance and Results Act (GPRA) for Best Practices Programs. This instrument asks about participant satisfaction with the training and its components. As per SAMHSA requirements, it is only collected at one time point, immediately post the ‘live’ DATA waiver training component. Analyses conducted were descriptive statistics (counts/frequencies).
- Personal Impact Survey. Administered immediately after the GPRA survey was the Personal Impact Survey. This was developed by the training initiative leadership to capture learners’ beliefs regarding changes in their knowledge, perception of OUD, and motivation to treat patients with OUD. Therefore, this survey was collected only immediately post ‘live’ DATA waiver training component. This survey asks learners to respond regarding knowledge, attitudes and future plans both before the training and immediately after the training, all within the post survey (see supplementary material). Paired t-tests were conducted to examine any statistically significant changes.
- Shadowing Experience Survey. The initiative leadership developed a local evaluation instrument to capture learner’s perceived changes in knowledge, understanding and views of OUD and MOUD treatment, as well as participant confidence, interest, motivation and plans to provide this treatment with patients in the future. This was collected within 30 days following clinical shadowing (see supplementary material). Analyses conducted were descriptive statistics (counts/frequencies).

## Results

### Study participants

Table 3 below shows demographics of the 306 learners that completed the DATA waiver training and evaluation components. Approximately two-thirds of participants were female (70%). Regarding race, the majority of participants selected White as their racial category (46%). The next largest group was persons selecting Hispanic/Latino as their race (23%), followed by 13% who chose multiple races, 12% who chose Asian, 3% who chose Native American, and 2% who selected Black or African American. Four respondents did not report race (Note that in New Mexico, some individuals report their race as Hispanic/Latino rather than selecting one of the federally prescribed racial categories). Regarding ethnicity, ninety-seven persons (32%) reported they were Hispanic/Latino. Learners were primarily medical students in their second post-graduate year, as this was the largest group of learners who were included as the focus population for the intervention. The intervention originally targeted only psychiatric nurse practitioners (PNPs). However, faculty members in the family nurse practitioner (FNP) program asked to have their learners included in the DATA waiver trainings. Their involvement was an unexpected positive outcome of this initiative and was the basis for the University’s subsequent proposal to expand this intervention for the entire University Nurse Practitioner program and was funded in October of 2021.

**Table 3:** Participant Demographics for DATA Waiver Training Participation (N = 306)

Gender	
Male	85 (28%)
Female	213 (70%)
Missing	8 (2%)
**Race**	
White Hispanic*	142 (46%) 69 (23%)
Multiple races	40 (13%)
Asian	38 (12%)
Native American	8 (3%)
Black or African American Missing	5 (2%) 4 (1%)
**Hispanic/Latino**	
Hispanic/Latino	97 (32%)
Non-Hispanic/Latino	206 (67%)
Missing	3 (1%)
**Type of Learner**	
Medical Student	191 (62%)
Family Nurse Practitioner Student	56 (18%)
Psychiatric Nurse Practitioner Student	15 (5%)
Physician Assistant Student	29 (10%)
Missing	15 (5%)

*GPRA Survey and Personal Impact Survey*. These survey results show a high overall satisfaction with the DATA waiver training provided. This included satisfaction with the quality of training, the instructor, the material and the overall training experience (see Table 4). For the Personal Impact Survey (PIS), results showed self-reported improvement in all items including view of OUD as chronic condition, knowledge of OUD treatment, motivation to treat patients with OUD, and plans to obtain the DATA waiver to prescribe buprenorphine. P < .001. Internal reliability using Cronbach’s Alpha is 0.899 for the GPRA survey, and 0.784 for the Personal Impact Survey, demonstrating strong internal reliability for both surveys.

**Table 4:** GPRA and Personal Impact Survey (N = 306)

Training Satisfaction					
	Very Satisfied/ Satisfied	Neutral	Dissatisfied Very Dissatisfied		Missing Data
Satisfaction with quality of training	288 (94%)	13 (4%)	3 (1%)		2 (1%)
Satisfaction with quality of instructor	226 (74%)	41 (13%)	2 (1%)		37 (12%)
Satisfaction with quality of training material	278 (91%)	23 (7%)	3 (1%)		2 (1%)
Overall Satisfaction with training experience	280 (92%)	19 (6%)	6 (2%)		1 (0%)
**Personal Impact Survey: Knowledge and Attitudes (N = 306)**					
	Pre-mean	Post-mean		p Value	
View OUD as a chronic condition	4.11	4.62		< 0.001	
Knowledgeable about treatment for OUD	3.26	4.44		< 0.001	
Motivated to treat patients with OUD in future practice	3.67	4.41		< 0.001	
Plan on obtaining waiver in order to prescribe Buprenorphine	3.62	4.42		< 0.001	

*Shadowing Experience Survey*. Table 5 shows results from the Shadowing Experience Survey. Because of challenges with clinic-based shadowing due to COVID-19, only 98 of the 306 learners were able to complete shadowing. This included 79 MDs, 15 PAs and 4 Psychiatric NPs; representing 41% of MDs (79/191), 52% of PAs (15/29), and 26% Psychiatric NPs (4/15). Because of the limited shadowing opportunities due to COVID, we were not able to offer shadowing to any of FNPs. Results from the 98 respondents show that 94% agreed or strongly agreed that the shadowing experience helped them understand what it is like to provide buprenorphine treatment in practice and understand the rewards of this treatment for patients. 83% responded that shadowing improved their confidence to provide this treatment, and approximately three-quarters responded that they were interested in working in a practice with OUD patients (72%). About half of respondents (53%) planned to look for employment with opportunities to work with patients with OUD, and about half (54%) planned for a residency or employment with opportunities to continue learning about OUD treatment. Finally, about one-third of respondents (34%) reported knowing how to apply for the federally required xDEA license to prescribe buprenorphine for treatment of OUD. Cronbach’s Alpha was 0.873 demonstrating strong internal reliability for the Shadowing Experience Survey.

**Table 5:** Shadowing results (N = 98)

Topic	Strongly Agree/ Agree	Neutral	Strongly Disagree/Disagree	Missing Data
	N (%)	N (%)	N (%)	
Improved understanding of what it is like to provide buprenorphine for OUD in practice	92 (94%)	3 (3%)	3 (3%)	0 (0%)
Improved understanding of rewards for patients for providing OUD buprenorphine treatment	92 (94%)	3 (3%)	3 (3%)	0 (0%)
Feel more confident in ability to treat patients with OUD	81 (83%)	11 (11%)	6 (6%)	0 (0%)
Increased interest in working in a clinical practice with patients with OUD	70 (72%)	19 (19%)	9 (9%)	0 (0%)
Plan for a residency or employment with opportunities to continue learning about OUD treatment	53 (54%)	32 (33%)	13 (13%)	0 (0%)
Plan to look for employment with opportunities to work with patients with OUD	52 (53%)	35 (36%)	11 (11%)	0 (0%)
Know how to apply for xDEA license*	33 (34%)	23 (23%)	38 (39%)	4 (4%)

*Two learners did not respond to this question

## Discussion

Our *University Providers Clinical Support System Training Initiative* was developed to prepare medical, psychiatric nurse practitioner and physician assistant students to prescribe buprenorphine for treatment of opioid use disorder (OUD) post-graduation. Our expected outcomes were to improve learners’ perceived knowledge, skills and attitudes about OUD buprenorphine treatment, positively impact their plans to provide this treatment post-graduation, and ensure they completed the DATA waiver training that enables them to prescribe buprenorphine to 30 or more active patients with OUD. Results from our evaluation show that the objectives of our program were met, with overall positive outcomes including perceived improvements in knowledge, better understanding of the benefits of this treatment for patients, improved attitudes toward persons with OUD, and increased motivation to provide this treatment post-graduation. Learners were very satisfied with the training program with over eighty-percent of learners reporting the program helped them feel more confident in providing this treatment.

The overall positive outcomes from this study align with findings from other medical student training initiatives on buprenorphine for treatment of OUD. For example, Zerbo and colleagues implemented a DATA waiver training with 4th year medical students with results showing 6-months post training statistically significant increases from their pre-tests in both knowledge and confidence with MOUD treatment, and an increase from baseline in reported likelihood to prescribe buprenorphine for OUD treatment [14]. Another program implementing the DATA waiver training with 4th year students showed similar increases in knowledge and feeling more prepared to treat patients with OUD using buprenorphine from pre to posttest [15]. Initiatives implementing the DATA wavier training with 3rd year medical students curricula shows similar positive results. For example, Riser and colleagues found statistically significant increases in knowledge of MOUD pharmacology, MOUD prescribing practices, and most students reported that the waiver training was relevant to their future practice [16].

Importantly, while integrating waiver training into learner curricula for long term sustainability is one approach, others also recommend simply encouraging leaners to complete the existing online waiver training available at PCSS.org as another cost-effective option [17]. This may be especially helpful for universities without grant funding or other resources to support curriculum modifications. One published example using this approach was Wayne State University’s School of Medicine in Michigan that encouraged 3rd year medical students to complete the DATA waiver training available online at PCSS.org. Their Pre-Post survey results showed increases in learners’ confidence in their ability to understand opioid use disorder, to prescribe buprenorphine, and increases in the percent of students who planned to provide this treatment in the future [18].

Our literature review did not reveal studies that compared training programs that offered only the DATA waiver and those that offered it alongside of in-person shadowing opportunities. In our initiative, shadowing was a funding requirement and our post Shadowing Experience survey shows overall positive responses especially in increasing understanding of OUD, benefits to patients and provider confidence, but somewhat less on future employment plans. Therefore, is not clear exactly what the impact of shadowing may provide learners in the long term. Future research that compares outcomes between various program types (i.e., those with and without shadowing; those imbedded in curriculums vs. those simply directing learners to PCSS.org) and those that included longer follow up (e.g. in the first few years of the learners independent practice) will be helpful in determining the most effective and efficient buprenorphine training programs.

Learner responses in our study indicated ways our initiative could be improved. For example, to address confidence and plans to provide this care in practice, we are working with our clinic partners to expand the shadowing component to increase experience with the treatment and help build confidence, adding additional course time for debriefing after shadowing to address concerns or lingering questions that may erode confidence. We also continue to collaborate with our state’s MOUD ECHO program to provide learners with additional exposure to practicing providers using buprenorphine for OUD, and to see that there are ongoing training and support mechanisms in place to guide them in starting this practice. Also, to help learners know how to apply for and obtain the xDEA license, one of our easiest but incredibly important improvements, we have created a simple “how to” form with step-by-step instructions and have a recorded webinar of this process.

Our evaluation results should be understood in the context of limitations. For instance, ability to participate in the in-person shadowing was limited due to COVID and in person restrictions that resulted in a smaller number of respondents for Shadowing Experience Survey. Another limitation is that the short-term nature of the results (post training, post shadowing) do not demonstrate changes in practice of this future workforce after graduation. However, the time and cost involved in conducting a longer follow up was not possible given the funding of the present training grant. Another limitation is that we did not use an objective measure of knowledge change. The post-test nature of our surveys includes potential participant bias where learners may wish to demonstrate improvements in self-reported knowledge. We attempted to reduce this bias by having students complete surveys anonymously.

## Conclusion

The results of our study contribute to the growing case for providing buprenorphine education in pre-licensure education in medical schools, physician assistant programs and nurse practitioner programs. Whether developing and embedding a full DATA waiver program and shadowing experience in pre-licensure medical, physician assistant, and nurse practitioner education curricula, as in our institution, or opting for a less intensive approach like those discussed above, educating every learner in MOUD treatment is a key effort in tacking the opioid crisis. Data suggests these approaches can help build a workforce of providers with more knowledge of MOUD, less stigma toward persons with OUD, and with shadowing components, give learners experience with providing this care directly. The main message from our study, as with other published initiatives to train medicals students on MOUD and perspectives from medical leaders, is that universities can expect to enhance their learners’ knowledge, confidence and plans to use buprenorphine treatment by implementing buprenorphine training programs. This can be accomplished through curriculum modifications, accessing the available online PCSS option, or both, and can be a critical step in addressing the lack of educated and willing providers to treat OUD.

## Electronic supplementary material

Below is the link to the electronic supplementary material.

Supplementary Material 1

Supplementary Material 2

## Data Availability

The datasets used and/or analyzed during the current study are available from the corresponding author on reasonable request.

## Declarations

### Authors’ information

VW, JS, LF, AE, and JK supported this initiative through their leadership roles in health education programming at the University (e.g., Medical School, Physician Education, and Nursing). VW, JS, LF, AE, JK and SB also provide direct services to persons with opioid use disorder and serve as champions for this initiative. As described in the manuscript, developing this intervention and evaluation relied on the professional positions and personal commitment (“champions”) of these authors. SBS, TK and JS are non-clinical and work in research and evaluation in the field of substance use disorders, and opioid use disorder.
