# Supplementary material for: Evaluation and guide for embedding opioid use disorder education in health professions’ curricula
Source: BMC Med Educ. 2023 Mar 1;23:135. doi: 10.1186/s12909-023-04088-5 (PMC9975819; doi:10.1186/s12909-023-04088-5)
Supplement: Supplementary file 1 — Supplementary Material 1 [file 12909_2023_4088_MOESM1_ESM.pdf]

Title: Personal Impact Survey.

Explanation: This survey was used to capture learners' beliefs regarding changes in their knowledge, perception of Opioid Use Disorder (OUD), and motivation to treat patients with OUD.

1. Do you Agree to Participate in this Evaluation?

- [ ] Yes, I agree to participate in the study to assess the impact of the PCSS DATA 2000 Waiver training
- [ ] No, I don't agree to participate in the study to assess the impact of the PCSS DATA 2000 Waiver training

2. Date of Buprenorphine Waiver Training

Date / Time

Date

MM/DD/YYYY

3. Gender

- ○ Male
- ○ Female

4. Are you Hispanic or Latino?

- ○ Yes
- ○ No

5. What is your ethnicity? (check all that apply)

- [ ] White (non-Hispanic)
- [ ] Hispanic
- [ ] Native American
- [ ] Black or African American
- [ ] Asian
- [ ] Native Hawaiian or other Pacific Islander
- [ ] Alaska Native

6. Sexual orientation-Do you identify as:

- ○ Straight/Heterosexual
- ○ Bisexual
- ○ Gay
- ○ Lesbian
- ○ Transgender
- ○ Prefer not to say

7. Type of Student

- Medical Student
- Psychiatric Nursing Student
- Family Practice Nursing Student
- PA Student

8. For Medical Students-Year of Training

- 1st
- 2nd
- 3rd
- 4th

9. Name of Trainer

- [ ] Snehal Bhatt
- [ ] Lindsay Fox
- [ ] Vanessa Jacobsohn

10. The material presented in this training increased my knowledge of opioid use disorder and medication-assisted treatment

- Strongly agree
- Agree
- Neutral
- Disagree
- Strongly disagree

11. Because of the seminar, I plan on obtaining the Buprenorphine DATA Waiver

- Strongly Agree
- Agree
- Undecided
- Disagree Strongly
- Disagree

12. I plan to use the information from the training to treat opioid use disorder

- Strongly Agree
- Agree
- Somewhat Agree
- Disagree Strongly
- Disagree

13. List two things you will incorporate into your professional work as a result of this training.

14. Overall Satisfaction

	Very satisfied	Satisfied	Neutral	Dissatisfied	Very dissatisfied
How satisfied are you with the quality of this training?	○	○	○	○	○
How satisfied are you with the quality of the instructor Dr. Bhatt?	○	○	○	○	○
How satisfied are you with the quality of the instructor Dr. Stanton	○	○	○	○	○
How satisfied are you with the quality of the training materials?	○	○	○	○	○
Overall, how satisfied are you with your training experience?	○	○	○	○	○

15. Training Organization

	Strongly Agree	Agree	Neutral	Disagree	Strongly Disagree
The training class was well organized.	○	○	○	○	○
The material presented in this class will be useful to me in dealing with substance abuse.	○	○	○	○	○
The instructor was knowledgeable about the subject matter.	○	○	○	○	○
The instructor was well prepared for the course.	○	○	○	○	○
The instructor was receptive to participant comments and questions.	○	○	○	○	○
I would recommend this training to a colleague	○	○	○	○	○

16. Training Relevance

	Strongly Agree	Agree	Neutral	Disagree	Strongly Disagree
I am currently effective when working in this topic area.	◯	◯	◯	◯	◯
The training enhanced my skills in this topic area	◯	◯	◯	◯	◯
The training was relevant to my career	◯	◯	◯	◯	◯
I expect to use the information gained from this training	◯	◯	◯	◯	◯
I expect this training to benefit my patients	◯	◯	◯	◯	◯
This training was relevant to substance abuse treatment	◯	◯	◯	◯	◯

17. How useful was the information you received from the instructor?

- ◯ Very useful
- ◯ Useful
- ◯ Neutral
- ◯ Not useful
- ◯ Not Applicable

18. Please rate your agreement with the following statements **BEFORE** attending today's Buprenorphine training

	Strongly Agree	Agree	Neutral	Disagree	Strongly Disagree
I viewed opioid use disorder as a chronic disorder, similar to other chronic conditions such as diabetes or major depressive disorder.	◯	◯	◯	◯	◯
I was knowledgeable about treatment options for opioid use disorder.	◯	◯	◯	◯	◯
I was motivated to treat patients with opioid use disorder in my future practice	◯	◯	◯	◯	◯
I planned on obtaining a Buprenorphine waiver in order to prescribe buprenorphine for patients with opioid use disorder.	◯	◯	◯	◯	◯

19. Please rate your agreement with the following statements **AFTER** attending today's Buprenorphine training

	Strongly Agree	Agree	Neutral	Disagree	Strongly Disagree
I view opioid use disorder as a chronic disorder, similar to other chronic conditions such as diabetes or major depressive disorder.	◯	◯	◯	◯	◯
I am knowledgeable about treatment options for opioid use disorder.	◯	◯	◯	◯	◯
I am motivated to treat patients with opioid use disorder in my future practice	◯	◯	◯	◯	◯
I plan on obtaining a Buprenorphine waiver in order to prescribe buprenorphine for patients with opioid use disorder.	◯	◯	◯	◯	◯

20. Please describe the Strengths of the training

21. Please provide suggestions for training Improvement

22. The seminar was effective and helped me meet the rotation objectives

- Strongly Agree
- Agree
- Undecided
- Disagree Strongly
- Disagree

23. Please Provide

First Name

Email Address

Phone Number
