# Supplementary material for: Evaluation and guide for embedding opioid use disorder education in health professions’ curricula
Source: BMC Med Educ. 2023 Mar 1;23:135. doi: 10.1186/s12909-023-04088-5 (PMC9975819; doi:10.1186/s12909-023-04088-5)
Supplement: Supplementary file 2 — Supplementary Material 2 [file 12909_2023_4088_MOESM2_ESM.pdf]

Title: Shadowing Experience Survey. Explanation: To capture learner's perceived changes in knowledge, understanding and views of OUD and MOUD treatment, as well as participant confidence, interest, motivation and plans to provide this treatment with patients in the future.

1. Date

Date / Time

Date

MM/DD/YYYY

2. Gender

- ◯ Male
- ◯ Female
- ◯ Other

3. Please specify other gender:

4. Type of Student

- ◯ Medical Student
- ◯ Nursing Student
- ◯ Physician Assistant Student

5. For Medical Students- year of training

- ◯ 1st
- ◯ 2nd
- ◯ 3rd
- ◯ 4th

6. Please rate the following: After completing the DATA waiver and Shadowing experience...

	Strongly Agree	Agree	Neutral	Disagree	Strongly Disagree	Not Applicable
I know how to apply for my XDEA number and obtain my license to prescribe buprenorphine for opioid use disorder.	○	○	○	○	○	○
My understanding of what it is like to provide buprenorphine for opioid use disorder in practice has improved	○	○	○	○	○	○
I have a better understanding of the rewards for patients of providing treatment for opioid use disorder using buprenorphine	○	○	○	○	○	○
I am more interested in providing buprenorphine treatment for persons with opioid use disorder.	○	○	○	○	○	○
I am more interested in working in a clinical practice with patients who have opioid use disorder	○	○	○	○	○	○
I feel more confident in my ability to treat patients with opioid use disorder using buprenorphine	○	○	○	○	○	○
I plan to look for a residency program or employment that provides me with opportunities to learn more about the treatment of opioid disorder using buprenorphine	○	○	○	○	○	○
I plan to look for employment that provides me with the opportunity to work with patients with opioid use disorder.	○	○	○	○	○	○

7. Please rate you agreement with the following statements After DATA Waiver and Shadowing experience

	Strongly Agree	Agree	Neutral	Disagree	Strongly Disagree
I view opioid use disorder as a chronic disorder, similar to other chronic conditions such as diabetes or major depressive disorder.	○	○	○	○	○
I am knowledgeable about treatment options for opioid use disorder.	○	○	○	○	○
I am motivated to treat patients with opioid use disorder in my future practice.	○	○	○	○	○
I plan on obtaining a buprenorphine waiver in order to prescribe buprenorphine for patients with opioid use disorder.	○	○	○	○	○

8. Overall, how would you describe your shadowing experience?

Excellent	Above Average	Average	Below Average	Very Poor
○	○	○	○	○

9. Satisfaction with Shadowing

	Strongly Agree	Agree	Neutral	Disagree	Strongly Disagree	Not Applicable
I feel that the 4 hours of shadowing provided me with the clinical experience I needed	○	○	○	○	○	○
The clinical setting was conducive to learning the role of a MAT provider	○	○	○	○	○	○
The clinical staff were receptive to my questions	○	○	○	○	○	○

10. What appeals to you the most about obtaining your xDEA?

11. What is least appealing about obtaining your xDEA

12. Please share any additional comments about this training program

13. Was there any information/ experience you wish you would have received that you did not receive?
